# Machine learning with autophagy-related proteins for discriminating renal cell carcinoma subtypes

**DOI:** 10.1038/s41598-020-57670-y

**Published:** 2020-01-20

**Authors:** Zhaoyue He, He Liu, Holger Moch, Hans-Uwe Simon

**Affiliations:** 1 Institute of Pharmacology, University of Bern, Bern, Switzerland; 2 University Institute of Clinical Chemistry, Inselspital, Bern University Hospital, University of Bern, Bern, Switzerland; 3 Department of Pathology and Molecular Pathology, University and University Hospital Zurich, Zurich, Switzerland; 4 Department of Clinical Immunology and Allergology, Sechenov University, Moscow, Russia

**Keywords:** Tumour biomarkers, Kidney diseases

## Abstract

Machine learning techniques have been previously applied for classification of tumors based largely on morphological features of tumor cells recognized in H&E images. Here, we tested the possibility of using numeric data acquired from software-based quantification of certain marker proteins, i.e. key autophagy proteins (ATGs), obtained from immunohistochemical (IHC) images of renal cell carcinomas (RCC). Using IHC staining and automated image quantification with a tissue microarray (TMA) of RCC, we found ATG1, ATG5 and microtubule-associated proteins 1A/1B light chain 3B (LC3B) were significantly reduced, suggesting a reduction in the basal level of autophagy with RCC. Notably, the levels of the ATG proteins expressed did not correspond to the mRNA levels expressed in these tissues. Applying a supervised machine learning algorithm, the K-Nearest Neighbor (KNN), to our quantified numeric data revealed that LC3B provided a strong measure for discriminating clear cell RCC (ccRCC). ATG5 and sequestosome-1 (SQSTM1/p62) could be used for classification of chromophobe RCC (crRCC). The quantitation of particular combinations of ATG1, ATG16L1, ATG5, LC3B and p62, all of which measure the basal level of autophagy, were able to discriminate among normal tissue, crRCC and ccRCC, suggesting that the basal level of autophagy would be a potentially useful parameter for RCC discrimination. In addition to our observation that the basal level of autophagy is reduced in RCC, our workflow from quantitative IHC analysis to machine learning could be considered as a potential complementary tool for the classification of RCC subtypes and also for other types of tumors for which precision medicine requires a characterization.

## Introduction

Autophagy, a dynamic catabolic process, characterized by the degradation of cellular contents in double membrane-forming autophagosomes, is well known for its essential roles in regulating cellular homeostasis^1,2^. The class III PI3K and mTOR pathways, as well as the so-called ATGs are key regulators of autophagy^3^. Although kept at relatively low level, autophagy can be induced by diverse stresses, e.g. growth factor withdrawal or administration of anti-cancer drugs. Interestingly, however, autophagy induced by anti-cancer therapy has two sided effects, either protecting cancer cells from drug-induced cell death, or promoting drug-induced cell death by inducing “autophagic cell death” in cancer cells^3,4^. With IHC staining of ATG5 and LC3 in paraffin sections derived from primary melanomas and testicular germ cell tumors, we found that both ATG5 expression and autophagy generally are downregulated in these cancer patients^5,6^. In a mouse model of lung cancer, it has been found that a deficiency in autophagy accelerates tumor initiation, favors, however, overall survival once a tumor is formed^7^.

RCC is composed of heterogeneous neoplastic cells arising from renal tubular epithelial cells and is the most lethal malignant urological tumor. The most frequent histological subtypes accounting for more than 90% of all RCCs are clear cell renal cell carcinomas (ccRCC), papillary renal cell carcinomas (pRCC) and chromophobe renal cell carcinomas (crRCC)^8–10^. Research efforts to identify molecular markers for discriminating among these subtypes suggest that histone methyltransferases and microRNA-145 may have diagnostic value for discrimination of certain subtypes of RCC^11,12^.

In contrast to the vast amounts of literature investigating the role of autophagy in anti-cancer therapy, research on the significance of the basal level of autophagy in tumors remains rare. In case of RCC, it has been reported that a combined analysis of several autophagy markers could contribute to a prediction of postoperative disease recurrence in patients with ccRCC^13^. In RCC with characteristic cytoplasmic inclusions composed of protein aggregates and peroxisomes, somatic mutations or high frequencies of genetic variations in *ATG7*, *ATG5* and *ATG10* were found to be associated with the formation of these inclusions, suggesting a possible defect in autophagy in these patients^14^.

Machine learning algorithms have been widely applied for the recognition of nuclei, for detection of tissue segmentation^15^, for breast cancer diagnosis^16^, and for classification and mutation prediction in lung cancer^17^. In contrast to the raw medical images as input data for machine learning used to now, the application of numeric data generated from the quantification of immunohistochemical images for machine learning has remained rare. Here, we have selected a simple and fast classification algorithm, the K-Nearest Neighbor (KNN) algorithm, for discrimination among RCC subtypes. Compared to other algorithms, KNN is easy to understand and to implement. For machine learning with KNN, we used the normalized Integrated Optical Density (IOD) values obtained from IHC staining of ATG proteins as features or variables and the patients diagnosed with different subtypes as observations. In this study, we show a significant downregulation of ATG1, ATG5 as well as LC3B in RCC by IHC staining followed by software-based quantification of the IODs of these autophagy marker proteins, suggesting a reduced basal level of autophagy in RCC patients *in vivo*. Our machine learning algorithm with the IODs thus obtained suggested that LC3B provided a strong measure for discriminating clear cell RCC (ccRCC). ATG5, and sequestosome-1 (SQSTM1 or p62) could be used for classification of chromophobe RCC (crRCC). A combination of ATG1, ATG16L1, ATG5, LC3B and p62, was able to discriminate among normal tissue, crRCC and ccRCC. Thus, our work indicates the potential for bioinformatics approaches in tumor classification based on the expression levels of certain ATGs in RCC.

## Materials and Methods

### Patient cohort and TMA

The TMA containing 237 RCCs from untreated patients and 18 normal kidney tissues from healthy donors was constructed by the Department of Pathology and Molecular Pathology, University and University Hospital Zurich. All methods were performed in accordance with the relevant guidelines and regulations as previously described^18^.

### IHC

IHC was performed as previously described^5^. Briefly, paraffin-embedded TMAs were deparaffinized, rehydrated, and subjected to antigen retrieval. The Dako REAL Detection System, Alkaline Phosphatase/RED kit was applied to stain the tissue sections according to the instructions provided (K5005, Dako). The following antibodies were used: anti-ATG1 (AP8104b, Abgent), anti-ATG16L1 (LS-B2723, Lifespan Biosciences), anti-ATG5 (11C3, Nanotools), anti-LC3B (023–100, Nanotools) and anti-p62 (P0067, Sigma).

### Quantification of the staining intensity

The staining intensities of the proteins of interests were quantified as IODs with Image Pro Plus as described^5^.

### Statistical analysis

The statistical analysis was performed with the unpaired Student *t*-test or ANOVA followed by the Bonferroni test for multiple comparisons as indicated. p < 0.05 is considered as statistically significant.

### Machine learning with R

All data analysis was performed using the R language, including data cleaning and machine learning^19^. The data were centered and scaled by normalization with mean and SD values before submission to the machine learning algorithm. The K-Nearest Neighbor (KNN) algorithm was selected for machine learning^20^. Upon stratified sampling, 55% of the data were used for training and the remaining 45% were used for testing. Receiver Operating Characteristics (ROC) curves and Area Under Curve (AUC) values were calculated using the “pROC” package. Training data were used to build the KNN model with optimal K values determined by a 4-fold cross validation. The cross validation was performed using 4 subsets of training data generated by stratified sampling. Three of the subsets were used for training and one was used for testing. This process iterated 4 times until all subsets were tested for validating and optimizing the learning model. Next, testing data were passed to the model for prediction. The performance of the KNN models was measured by Accuracy and Kappa or Cohen’s Kappa values^21^ using the “caret” package. Accuracy showed the percentage of correctly classified instances out of all instances. Kappa is calculated by (p_o _− p_e_)/(1 − p_e_), while p_o_ is the accuracy and p_e_ is the hypothetical probability of chance agreement. Kappa represented the accuracy normalized at the baseline of random chance on the dataset, serving to indicate the statistical inter-rater agreement.

### Ethical approval

Our retrospective study fulfilled the legal conditions according to Article 34 of the Swiss Law “Humanforschungsgesetz (HFG)”, which, in exceptional cases, allows the use of biomaterial and patient data for research purposes without informed consent, if i) it is impossible or disproportionately difficult to obtain patient consent; ii) there is no documented refusal; iii) research interests prevail over the individual interest of a patient. That the legal conditions of this study were abided to was reviewed and approved by the Ethics Commission of the Canton Zurich (KEK-ZH StV 25–2008, BASEC-Nr. PB_2016-02377).

## Results

### Reduced expression of ATG1, ATG5 and LC3B in RCC

**Figure 1:**
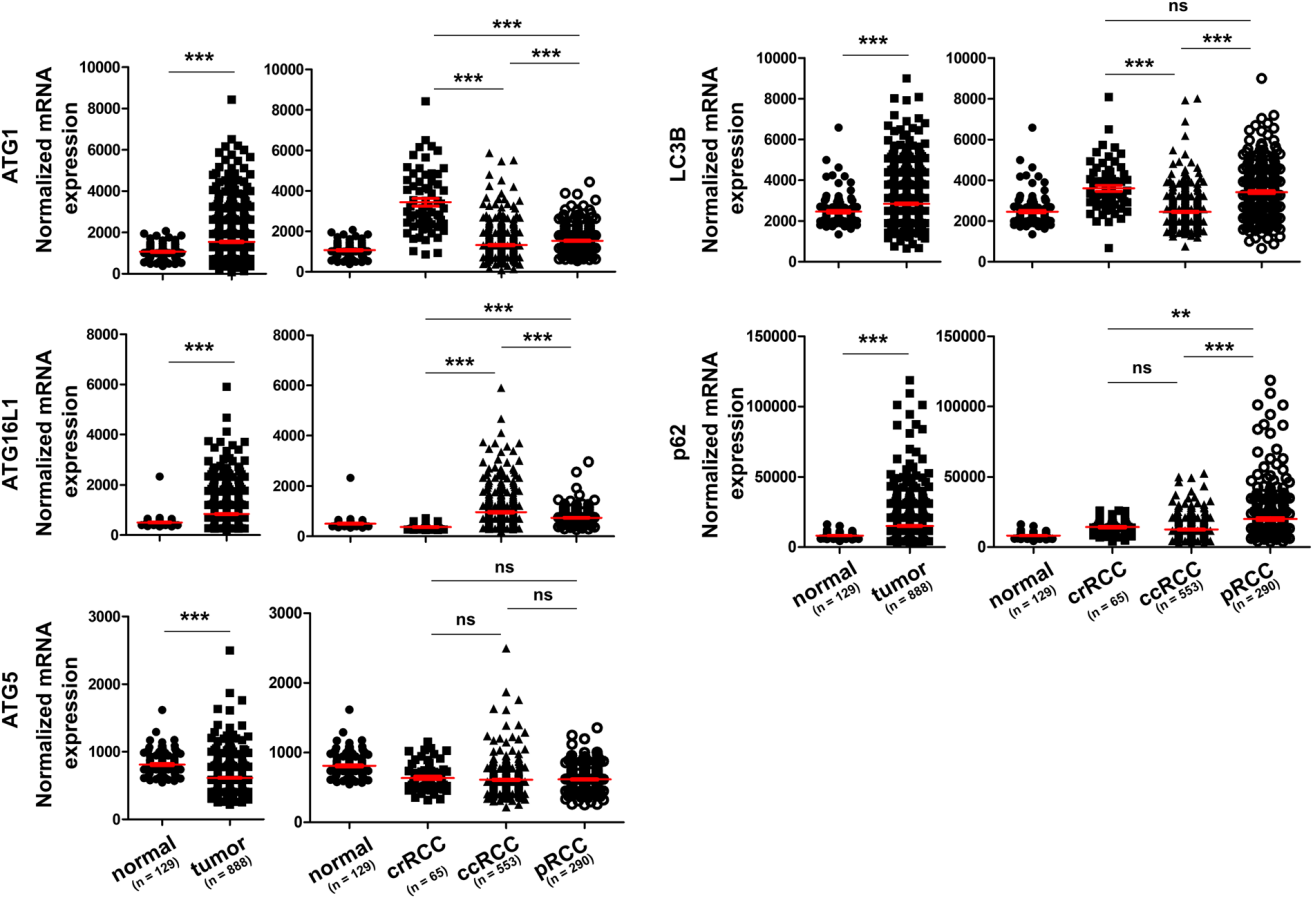
The mRNA expression of key *ATGs* in RCC. The mRNA expression data for *ATGs* in RCC was obtained from the TCGA consortium data base presented here as normalized mRNA expression with means and SEMs. **p < 0.01; ***p < 0.001; ns, not significant.

Based on the mRNA expression data available from The Cancer Genome Atlas (TCGA) consortium (https://cancergenome.nih.gov/), we compared the levels of the indicated *ATGs* in different subtypes of RCC and found that the transcripts of *ATG1*, *ATG16L1*, *LC3B* and *p62* were increased in the tumor compared with the normal tissues (Fig. 1). However, the *ATG5* mRNA level was decreased in the tumor compared with that found in the normal tissues (Fig. 1). Except *ATG5*, which showed similar levels among all RCC subtypes, the rest of the *ATGs* showed differential expression within RCC subtypes (Fig. 1).

**Figure 2:**
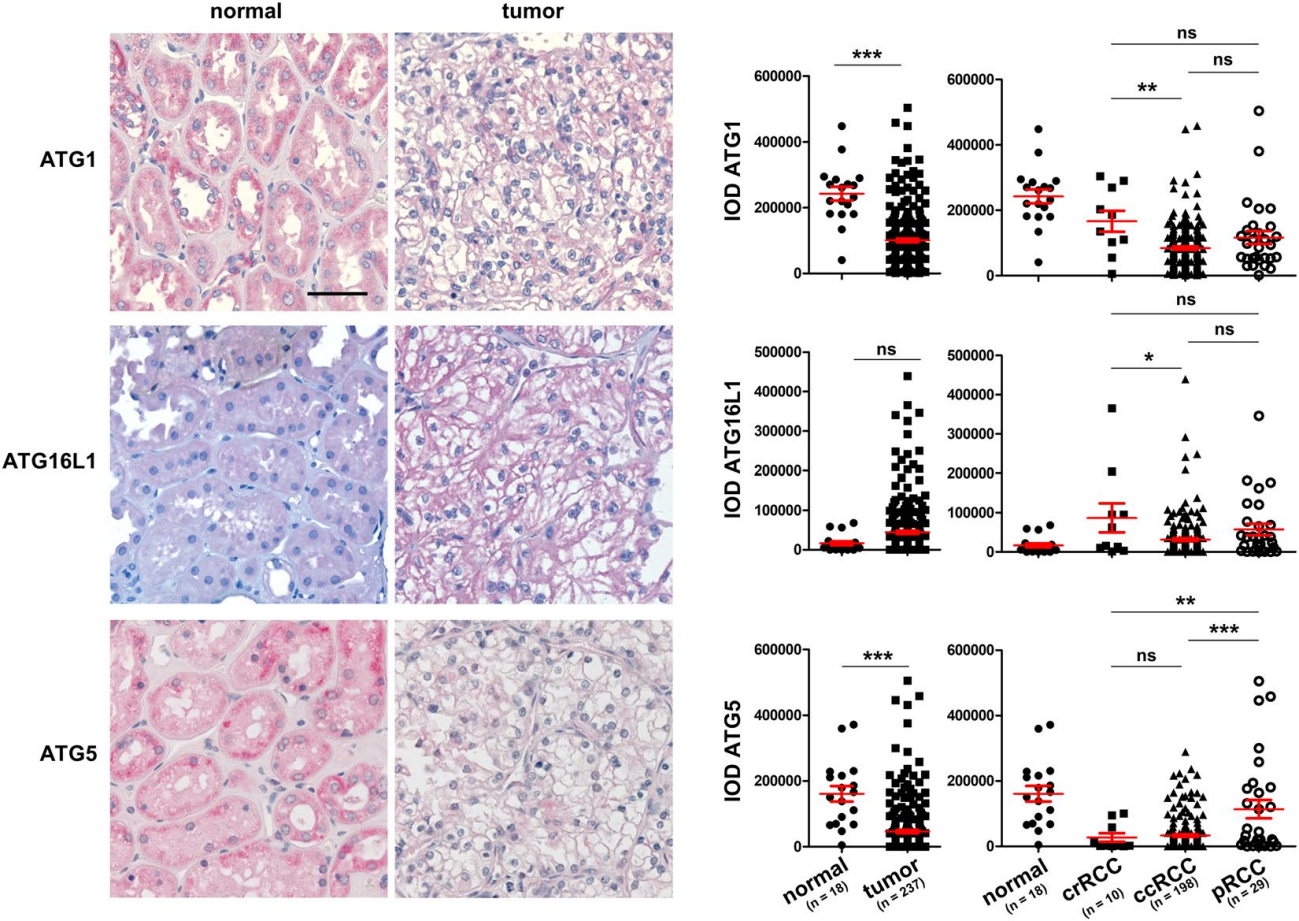
IHC staining of ATGs and their quantification. Left panel: Representative images of IHC for ATG1, ATG16L1 and ATG5. Right panel: The IODs quantified with Image Pro Plus software are presented as means and SEMs.

**Figure 3:**
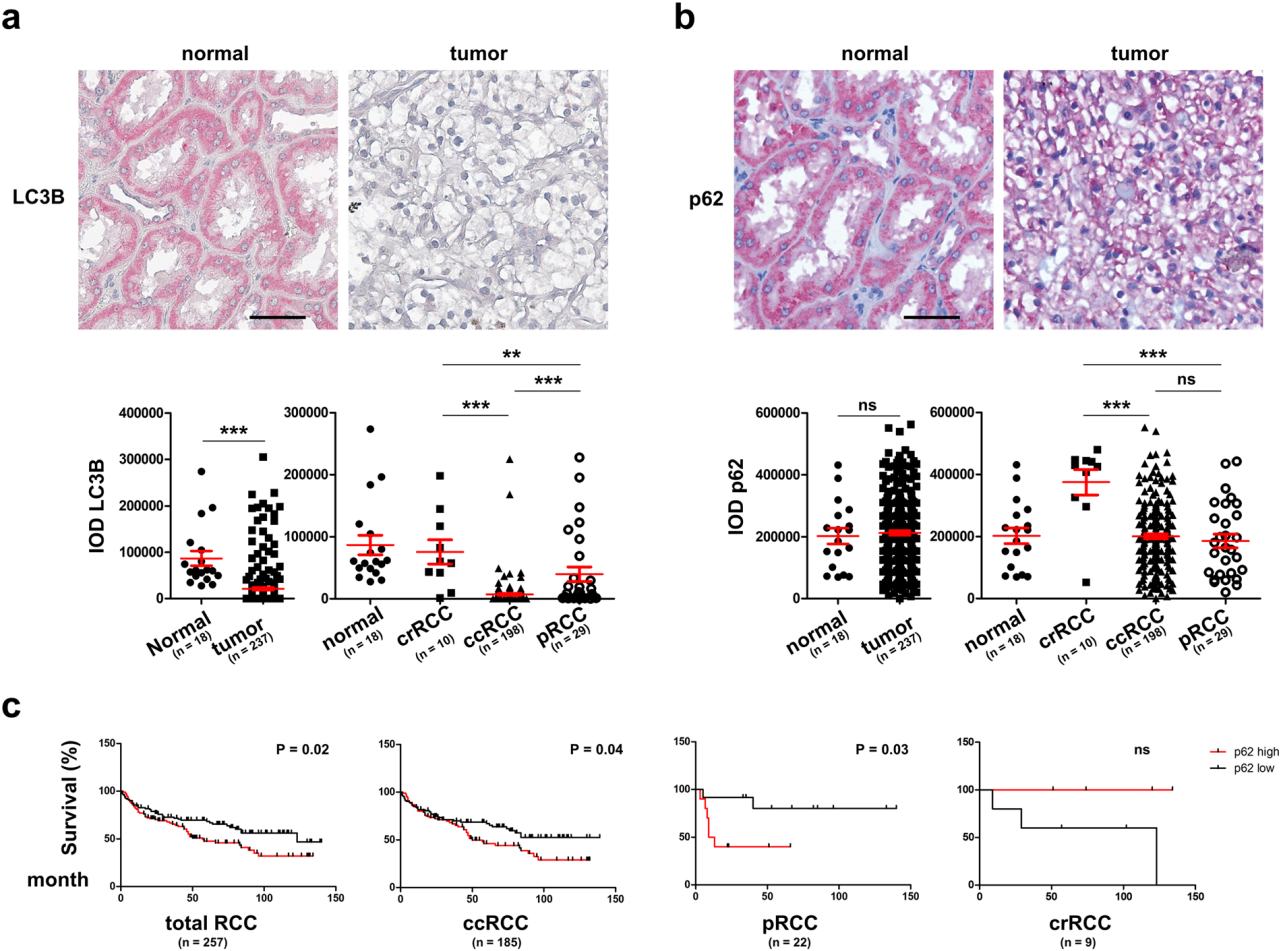
(**a**,**b**), Representative images of IHC staining of LC3B and p62 and their quantification presented as means and SEMs. (**c**) Survival curves of the RCC patients with high and low levels of p62. Scale bars: 50 µm. Statistical analysis among RCC subtypes was performed with ANOVA followed by the Bonferroni test for multiple comparisons. *p < 0.05; **p < 0.01; ***p < 0.001; ns, not significant.

Using TMAs containing 237 RCCs and 18 normal kidney tissues, however, we found a reduction in the protein expression of ATG1 and ATG5 in the tumor compared with the normal tissues by IHC staining (Fig. 2). No difference in ATG16L1 protein was detected between normal and the tumor tissues (Fig. 2). Within RCC subtypes, ATG1 and ATG16L1 are differentially expressed between crRCCs and ccRCCs, whereas ATG5 expression differed between crRCCs and pRCCs as well as between ccRCCs and pRCCs (Fig. 2). Staining LC3B, an autophagosome marker protein^3^ revealed a significant reduction in LC3B in the tumor compared to the normal tissues (Fig. 3a), suggesting a reduced basal level of autophagy in RCC. Interestingly, LC3B showed differential expression among all three subtypes of RCCs (Fig. 3a). Although, p62, which is degraded through the process of autophagy^3^ does not show differential expression between the normal and the tumor tissues, its expression differed between crRCCs and ccRCCs as well as between crRCCs and pRCCs (Fig. 3b). Interestingly, RCCs, ccRCCs and pRCCs with low p62 expression showed better survivals than those with high levels of p62 (Fig. 3c), suggesting the prognostic value of p62 probably due to its autophagy-unrelated functions. However, other investigated ATGs did not correlate with patient survival (data not shown). In contrast to mRNA analysis, showing an overall increase in the gene expression of the *ATGs* (Fig. 1), our evaluation of protein expression by corresponding *ATGs* suggested however, that autophagy was decreased in RCCs (Figs. 2 and 3). Often mRNA levels of a gene do not correlate with that of the protein expression due to e.g. regulation of mRNA stability and/or translational repression. It has been shown that *ATG1* and *LC3* mRNAs are stabilized by repression of protein synthesis, thus serving as a pool in order to rapidly replenish ATG proteins required for starvation-induced autophagy^22^. This may explain at least partially the discrepancy between the TCGA mRNA expression data and our protein expression results. Therefore, it is essential to evaluate the level of autophagy based on protein expression rather than mRNA expression of essential *ATGs*.

### Autophagy and specific ATGs are adequate predictive markers for RCC subtypes

**Figure 4:**
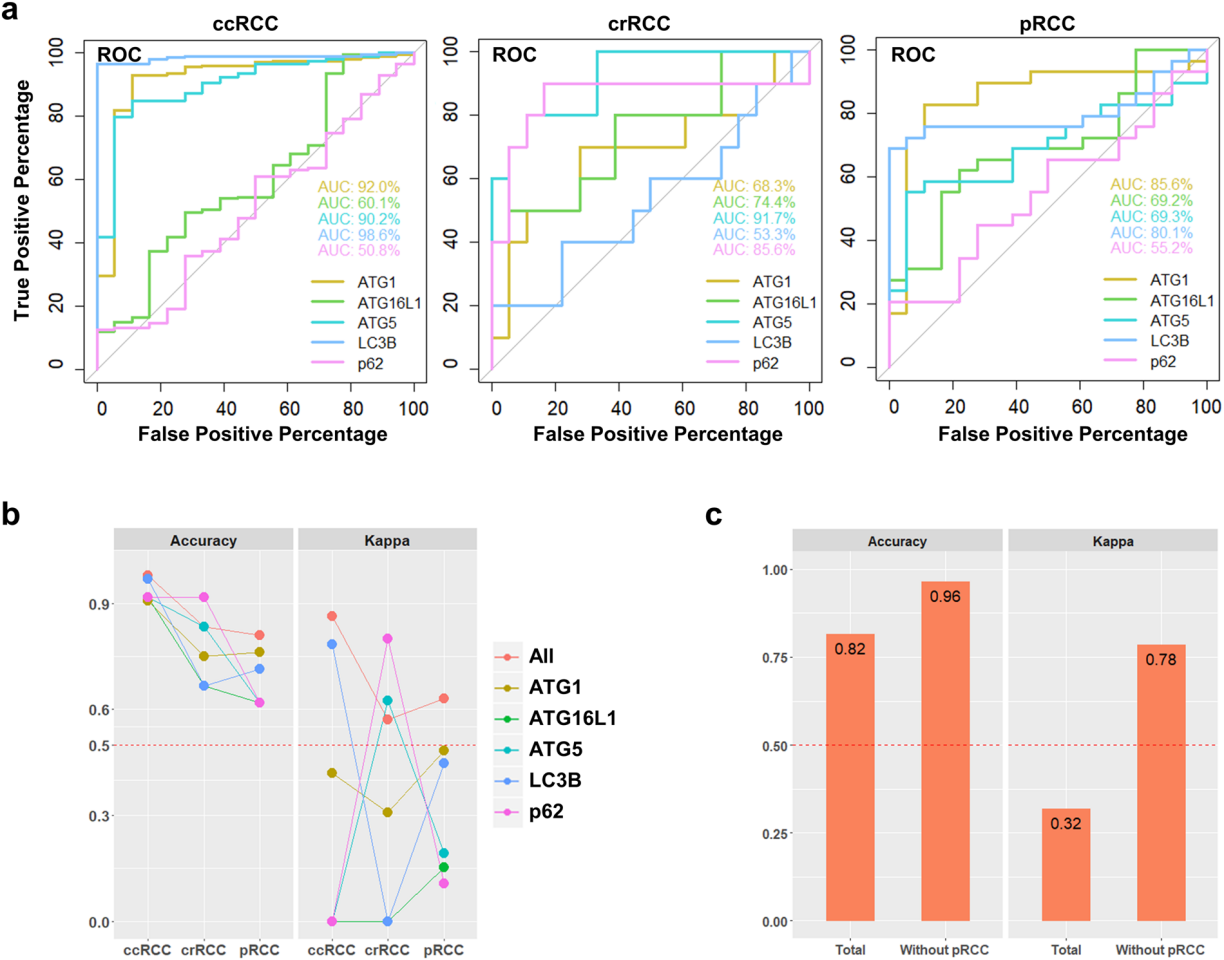
Machine learning to distinguish among RCC subtypes. (**a**) The ROC curves are shown for 3 tumor subtypes containing normal tissue. AUC values from different proteins are also presented on the plot. (**b**) Accuracy and Kappa values of the indicated ATGs or all 5 together (All) for discriminating RCC subtypes are presented. (**c**) Accuracy and Kappa values are presented as a measure of the performance of the machine learning algorithm with the total experimental data compared to the manipulated data excluding pRCC.

To evaluate whether autophagy and ATGs can be good measures for discriminating RCC subtypes, we applied the IODs of the corresponding ATGs to the KNN algorithm. To investigate the classification potential of each protein in different tumor subtypes against normal tissue, we plotted ROC curves and AUC values for each protein (Fig. 4a). In ccRCC, ATG1, ATG5 and LC3B showed high AUC values suggesting their potential to distinguish ccRCC from the normal kidney tissue. Similarly, ATG5, ATG16L1 and p62 showed high AUC values in subgroup of crRCC. In contrast, only ATG1 showed high AUC values in case of pRCC. To avoid imbalance among different sample sizes of our RCC subtypes and normal tissues, we used a stratified sampling approach and then divided the data obtained into two groups: 55% for training and the remaining 45% for testing. We performed a 4-fold cross validation with the training data to find the optimal K values to be used for building the KNN model (Supplementary Table 1). We tested our models using the testing data. Consistently with ROC curves, the proteins showed similar performance in distinguishing different subtypes from the normal tissue based on the Accuracy and Kappa values (Fig. 4b). Next, we combined all subgroups of RCC and normal tissue with values for all five proteins and repeated the same learning process to study the performance of our model in discrimination of the mixed tissues. Results of Accuracy and Kappa values showed that our study had a good performance, indicating that machine learning with the combination of these proteins could be used to distinguish different kidney tissues containing both tumors and normal tissues. Additionally, as most of the tested proteins could not differentiate pRCC from normal tissue, our model could even be improved if pRCC was not included (Fig. 4c), suggesting that evaluation of autophagy and/or ATGs are less sufficient for pRCC prediction as compared with other subtypes. In this case, other marker proteins, either related or unrelated to autophagy, need to be included in order to discriminate pRCC.

## Discussion

Although visual inspection of histopathological tissue samples by pathologists is still the standard approach to classification of tumor subtypes, recently developed computational approaches including automatic image processing and machine learning techniques may dramatically change the routine work flow of pathological diagnosis in the future. Despite the small sample size, we provided evidence that the quantified numeric data are also suitable for machine learning that does not require a sophisticated machine learning algorithm to discriminate RCC subtypes. Engaging machine learning in quantitative image analysis that allows an objective evaluation of the expression of the target proteins is rare. Our approach from IHC staining of ATGs, quantification of ATG expression to the application of machine learning algorithm, would assist pathologists and clinicians in patients’ classification and might contribute to targeted therapy. With increasing numbers of cases that can be used as training data, our protocol will be optimized to increase the recall and precision of the prediction, thus representing a quantitative means in precision medicine.

In this work, we have evaluated the expression of the key ATGs in RCCs by IHC and have quantified the obtained images that were later applied as numeric data to an R-based machine learning algorithm to classify the subtypes of RCCs. We have found a reduction in the basal level of autophagy in tumors as evidenced by reduced expression of ATG1, ATG5 and LC3B. Furthermore, LC3B provided a strong measure to discriminate ccRCC. ATG5 and p62 could be used for classification of crRCC. The combination of all these markers was able to predict normal tissue, crRCC and ccRCC, thus suggesting that the basal level of autophagy could be a potential measurement for RCC discrimination. Our workflow using quantified image analysis together with a machine learning algorithm may have clinical implications for modern pathology and precision medicine.

## Supplementary information

Supplementary Table 1.
