# Supplementary material for: Machine learning with autophagy-related proteins for discriminating renal cell carcinoma subtypes
Source: Sci Rep. 2020 Jan 20;10:720. doi: 10.1038/s41598-020-57670-y (PMC6971298; doi:10.1038/s41598-020-57670-y)
Supplement: Supplementary file 1 — Supplementary Table 1. [file 41598_2020_57670_MOESM1_ESM.docx]

**Supplementary Information**

**Machine learning with autophagy-related proteins for discriminating renal cell carcinoma subtypes**

**Zhaoyue He^1,2^, He Liu^1^, Holger Moch^3^ and Hans-Uwe Simon^1,4^**

^1^Institute of Pharmacology, University of Bern, Bern, Switzerland

^2^University Institute of Clinical Chemistry, Inselspital, Bern University Hospital, University of Bern, Bern, Switzerland

^3^Department of Pathology and Molecular Pathology, University and University Hospital Zurich, Zurich, Switzerland

^4^Department of Clinical Immunology and Allergology, Sechenov University, Moscow, Russia

**Correspondence**

Hans-Uwe Simon, Institute of Pharmacology, University of Bern, Inselspital, INO-F, CH-3010 Bern, Switzerland

Tel: +41 31 632 32 81

E-mail: [hans-uwe.simon@pki.unibe.ch](mailto:hans-uwe.simon@pki.unibe.ch)

| **Protein** | **ccRCC** | **crRCC** | **pRCC** |
| --- | --- | --- | --- |
| all | 7 | 5 | 9 |
| ATG1 | 7 | 9 | 9 |
| ATG16L1 | 5 | 9 | 9 |
| ATG5 | 7 | 5 | 5 |
| LC3B | 9 | 5 | 9 |
| p62 | 5 | 5 | 9 |

**Supplementary Table 1.** The optimal K values for KNN models were obtained via 4-fold cross validation with stratified sampling approach.
